# Delineating the interplay between oncogenic pathways and immunity in anaplastic Wilms tumors

**DOI:** 10.1038/s41467-023-43290-3

**Published:** 2023-11-30

**Authors:** Xiaoping Su, Xiaofan Lu, Sehrish Khan Bazai, Linda Dainese, Arnauld Verschuur, Benoit Dumont, Roger Mouawad, Li Xu, Wenxuan Cheng, Fangrong Yan, Sabine Irtan, Véronique Lindner, Catherine Paillard, Yves Le Bouc, Aurore Coulomb, Gabriel G. Malouf

**Affiliations:** 1 Department of Bioinformatics and Computational Biology, The University of Texas MD Anderson Cancer Center, Houston, TX USA; 2 Department of Cancer and Functional Genomics, Institute of Genetics and Molecular and Cellular Biology, CNRS/INSERM/UNISTRA, Illkirch, France; 3 Research Center of Biostatistics and Computational Pharmacy, China Pharmaceutical University, Nanjing, China; 4 Department of Pathology, Hôpital Armand Trousseau, Assistance-Publique Hôpitaux de Paris, Sorbonne Université, Paris, France; 5 UF Tumorothèque HUEP, Hôpital Armand Trousseau, Assistance-Publique Hôpitaux de Paris, Sorbonne Université, Paris, France; 6 Centre de Recherche Saint-Antoine (CRSA), INSERM, Sorbonne Université, UMR_S .938, Paris, France; 7 Department of Pediatric Oncology, Hôpital d’Enfants de La Timone, F-13005 Marseille, France; 8 Centre Léon Bérard, Institut d’Hématologie et d’Oncologie Pédiatrique (IHOPe), Lyon, France; 9 Department of Medical Oncology, Groupe Hospitalier Pitié-Salpêtrière, Assistance-Publique Hôpitaux de Paris, Paris, France; 10 Department of Pediaric Surgery, AP-HP, Hôpital Armand Trousseau, Sorbonne Université, Paris, France; 11 Department of Pathology, CHRU Strasbourg, Strasbourg, France; 12 Department of Pediatric Onco-hematology, CHRU Strasbourg, Strasbourg Université, Strasbourg, France; 13 Department of Medical Oncology, Institut de Cancérologie de Strasbourg, Strasbourg University, Strasbourg, France

**Keywords:** Paediatric cancer, Wilms tumour, Cancer genomics

## Abstract

Wilms tumors are highly curable in up to 90% of cases with a combination of surgery and radio-chemotherapy, but treatment-resistant types such as diffuse anaplastic Wilms tumors pose significant therapeutic challenges. Our multi-omics profiling unveils a distinct desert-like diffuse anaplastic Wilms tumor subtype marked by immune/stromal cell depletion, *TP53* alterations, and cGAS-STING pathway downregulation, accounting for one-third of all diffuse anaplastic cases. This subtype, also characterized by reduced CD8 and CD3 infiltration and active oncogenic pathways involving histone deacetylase and DNA repair, correlates with poor clinical outcomes. These oncogenic pathways are found to be conserved in anaplastic Wilms tumor cell models. We identify histone deacetylase and/or WEE1 inhibitors as potential therapeutic vulnerabilities in these tumors, which might also restore tumor immunogenicity and potentially enhance the effects of immunotherapy. These insights offer a foundation for predicting outcomes and personalizing treatment strategies for aggressive pediatric Wilms tumors, tailored to individual immunological landscapes.

## Abstract (web summary)

Treatment of diffuse anaplastic Wilms tumours (DAWT) remains a challenge. Here, the authors perform multi-omic analysis and identify a desert-like DAWT subtype accounting for one third of DAWT cases and suggest treating them with HDAC and/or WEE1 inhibitors.

## Introduction

Wilms tumor (WT) is a common pediatric solid tumor affecting the abdomen and closely linked to early nephrogenesis at both morphological and transcriptional levels^1,2^. The molecular drivers often mutated are related but not limited to the disruption of genetic pathways involved in the normal embryogenesis of the genitourinary tract, with around 40 genes reported to date^2^. Copy number alterations (CNAs), such as loss of chromosomes 1p and 16q or gain of 1q may contribute to tumor aggressiveness, but these do not provide insight into the mechanisms of tumor development or therapeutic vulnerabilities^2^.

The presence of anaplasia is a potent predictor of poor outcomes in patients with WT enrolled in the International Society of Paediatric Oncology (SIOP) (Europe and other countries) and the Children’s Oncology Group (COG) (North America and Canada) protocols^3,4^. While WT patients in European protocols receive preoperative chemotherapy, followed by surgery and then postoperative chemotherapy based on the histological risk assessment of the nephrectomy specimens, patients in American protocols are usually treated with surgery first. Previous studies have identified frequent WT mutations in genes such as *CTNNB1*^5,6^, *AMER1*^6–9^*, WT1*^6–9^, and *TP53*^10–14^. More recently, whole-exome sequencing (WES) identified recurrent mutations in the *SIX1* and *SIX2* pathways (18.1%) and in the *DROSHA/DGCR8* microprocessor genes (18.2%) in the blastemal type of WT^15^. Additionally, 11p15 abnormalities consisting of genetic or epigenetic defects are reported to be at the origin of 69% of WT^16,17^. *TP53* alterations, including mutations and/or deletions, have been found in 62.5% of diffuse anaplastic WT (DAWT) in a collaborative study involving SIOP (*n* = 8) and COG (*n* = 32) cases, and are associated with poor outcomes in terms of recurrence-free survival (RFS) and overall survival (OS)^10,18^. This study was consistent with a previous North American one which identified associations between *TP53* mutations, DAWT, and poor prognosis^12^. Moreover, some studies reported that mutant p53 suppresses innate immune signaling to promote tumorigenesis by interfering with the function of the cytoplasmic DNA sensing machinery cGAS-STING^19^.

While patients with focal anaplastic WT (FAWT) have identical survival to those without anaplasia, patients with DAWT are classified as high-risk tumors and therefore display poor outcomes^20^. Despite a high cure rate of over 90% with multimodal treatments including surgery and radio-chemotherapy, although this might come at the expense of high early and late toxicities^21^, subsets of patients whose tumors present diffuse anaplastic features or who experience recurrence after optimal multimodal therapies continue to pose therapeutic challenges^21,22^. One of the lingering questions in treating DAWT involves the as-yet-unexplored relationships among the transcriptome, genetic mutations, and the tumor microenvironment (TME)^23^.

In this work, we perform an integrative analysis of the genome-wide genetic, transcriptomic, and TME landscapes of DAWT relative to FAWT to delineate their prognosis and identify therapeutic vulnerabilities. We discover a desert-like DAWT subtype with global depletion of immune/stromal cells. We dissect the interplay between genetic and immune features, in particular loss of CD8+ cells and tumor intrinsic oncogenic pathways including histone deacetylase (HDAC) and DNA repair pathways. We validate our observation in an independent DAWT cohort. We also uncover that CD8+ and CD3 + T cells are capable of stratifying prognosis in pretreated anaplastic WTs rather than those without anaplasia. Finally, we provide evidence for the conservation of oncogenic pathways in anaplastic WT cell line models and identify therapeutic vulnerabilities through the use of HDAC and/or WEE1 inhibitors.

## Results

### Study population

In this study, we analyzed a total of 21 WTs with anaplastic features treated in France within the SIOP-2001 trial (Supplementary Data 1). The cohort comprised 9 DAWTs and 3 FAWTs, including 12 females and 9 males with a mean age of 5.4 years, ranging from 1.8 to 14.3 years. DNA and RNA of good quality and quantity were obtained from 12 and 10 patients, respectively. Additionally, matched germline DNA was obtained from adjacent kidney tissues in 12 cases. Consequently, WES was conducted on DNA from 12 paired WTs and their adjacent normal tissues, while the remaining 9 samples underwent targeted sequencing. RNA-seq was performed on 10 tumor samples.

### Genetic landscape of WT with focal and diffuse anaplasia

**Fig. 1:**
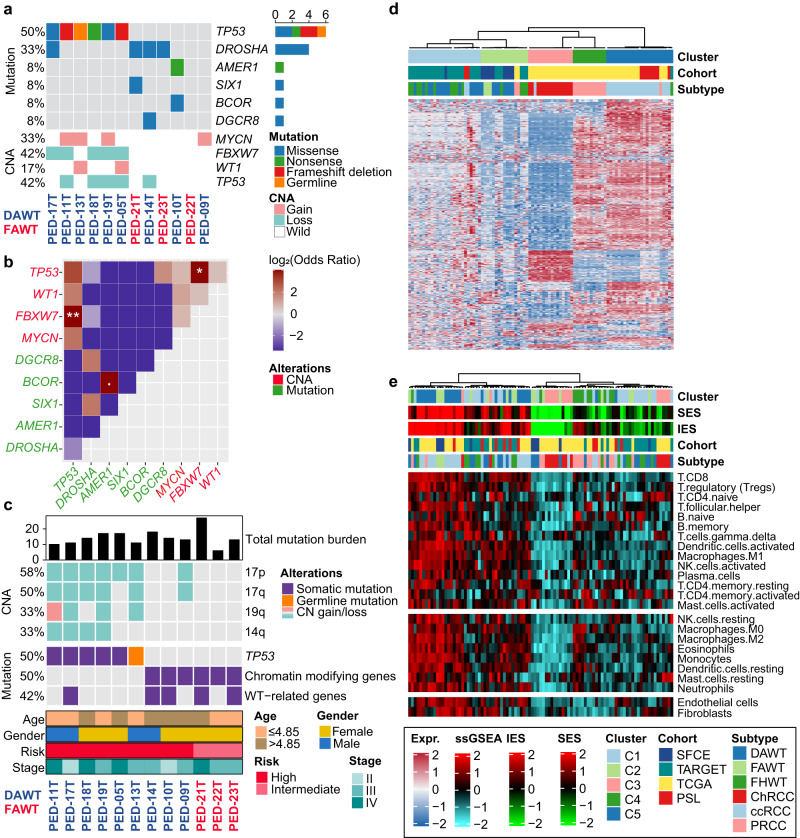
Genome-wide landscape of 12 Wilms tumors (WTs) identified by whole-exome sequencing and transcriptome pattern among 95 representative patients with four types of kidney cancers. **a** OncoPrint showing the mutation and copy number alteration (CNA) of Wilms tumor-related genes. **b** Genetic mutual exclusivity or cooccurrence in the SFCE-WT cohort using a one-side Fisher’s exact test (. *P* < 0.1, * *P* < 0.05, ** *P* < 0.01). **c** OncoPrint showing broad CNA (>25%) and mutations in a selection of frequently mutated genes arranged vertically by functional group. Total mutation burden was annotated at the top panel, and clinicopathological information was annotated in the bottom panel. **d** Heatmap showing the distinct expression pattern of four kidney cancers by unsupervised consensus clustering with most variable genes. **e** Two microenvironment subtypes with different immune/stromal infiltrations among 95 patients were identified by unsupervised clustering using curated signatures of 24 microenvironment cell types. Immune enrichment score (IES), stromal enrichment score (SES) and other cohort information were annotated at the top panel. SFCE Société Française du Cancer de l’Enfant, TARGET Therapeutically Applicable Research to Generate Effective Treatments, TCGA The Cancer Genome Atlas, PSL Pitié-Salpêtrière Hospital, DAWT diffuse anaplastic Wilms tumor, FAWT focal anaplastic Wilms tumor, FHWT favorable histology Wilms tumor, ChRCC chromophobe renal cell carcinoma, ccRCC clear cell renal cell carcinoma, PRCC papillary renal cell carcinoma. Source data are provided as a Source Data file.

Overall, 168 non-silent somatic single nucleotide variants (median 13, range: 6–27) were identified in the 12 anaplastic WTs assessed by WES (Fig. 1a; Supplementary Data 2). The mean nonsynonymous mutation rate was 0.27 mutations per Mb, with little variation (0.12–0.52). Of the mutations identified, 125 (74.6%) were not previously reported in the Catalogue of Somatic Mutations in Cancer (COSMIC) database, while 43 (25.4%) had been previously reported. The majority of the mutations were nonsynonymous single nucleotide substitutions (66.8%), followed by frameshift insertions and deletions (23.2%), stop gain (3.6%), splicing (2.8%), and non-frameshift deletions and insertions (2.8%). No difference was observed in terms of total mutation burden (TMB) between DAWT and FAWT (*P* = 0.93).

*TP53* and *DROSHA* were the only recurrent somatic mutations identified (Fig. 1a; Supplementary Data 2). Six out of nine DAWT (66.6%) harbored somatic (*n* = 5) and germline (*n* = 1) *TP53* mutations, while there were no mutations in FAWT. The germline mutation of *TP53* identified in PED-13T was identical to somatic mutations in PED-19T (Exon 6: [chr17_7574002; c.G548C; p.R183P; nonsynonymous SNV; G/C]). The mutation allelic fraction (MAF) of *TP53* in DAWT was particularly high, ranging from 85% to 97%, suggesting that *TP53* mutations may be a truncal event. In contrast, two DAWT (22%) and two FAWT (66.6%) had identical *DROSHA* hotspot mutations (E1147K) with MAF ranging from 1.8% to 42.7%. In addition to *DROSHA* mutations, cases harbored mutations in WT-related genes such as *BCOR* and *AMER1* (*n* = 1), *DGCR8* (*n* = 1), and *SIX1* (p.Q177R) (*n* = 1) (Supplementary Data 2). The MAF of *DGCR8* was 92.8%, suggesting that it may also be a truncal event. We also observed a significant co-occurrence pattern between CNA of *FBXW7* and *TP53* mutation (*P* = 0.008), as well as between CNA of *FBXW7* and CNA of *TP53* (*P* = 0.045) (Fig. 1b).

All WT without *TP53* mutations harbored mutations in chromatin-modifying genes (CMGs), including polycomb repressive complex genes *EZH2* (*n* = 1) and *BCOR* (*n* = 1), as well as *PROSER1* (*n* = 1), *KTM2C* (*n* = 1), *NASP* (*n* = 1), and *CDC73* (*n* = 1) (Fig. 1c; Supplementary Data 2). All of these mutations were predicted to result in deleterious proteins based on SIFT analysis^24^. We also observed that *TP53* mutation had significant co-occurrence with chromosome 17p loss, 17q loss, 14q loss, and 19q alteration (all, *P* < 0.05; Fig. 1c). In addition, we used GISTIC2.0 to examine focal copy-number variations in these tumors and found recurrent focal deletions in 11q14.3 and 16q22.1 (Supplementary Fig. 1a) and recurrent gain in 21q11.2 (Supplementary Fig. 1b).

### Assessment of *TP53* mutations and/or deletions in anaplastic WT

We then analyzed the *TP53* mutation status in the remaining cohort (eight DAWT and one FAWT) and discovered five additional DAWT with *TP53* mutations. Overall, 11 out of 17 (64.7%) DAWT harbored *TP53* mutations, while none of FAWT had them (Supplementary Data 1). Consistent with the high allelic *TP53* mutation fraction obtained, all cases with *TP53* mutations showed deletion of the second allele. Of note, all four patients with metastatic DAWT harbored *TP53* mutations and died with a median OS of 9.8 months (range: 5.9–12.3 months). In contrast, 7 out of 13 (53.8%) DAWT patients with localized disease (stage I-III) harbored *TP53* mutations; notably, three patients (42.9%) who harbored *TP53* mutation died within two years, showing marginally inferior OS as compared to those in *TP53* wild type (*n* = 6) (*P* = 0.08).

### Distinct transcriptome and immunity landscape among kidney cancers

We then asked whether WTs display different expression profiles compared to other kidney tumors, such as clear-cell, papillary, and chromophobe RCC. To this end, we performed unsupervised consensus clustering on a dataset of 95 kidney tumors (Supplementary Data 3). As expected, each histopathological subtype clustered separately from the others. In particular, WTs split into two clusters, one of which contained all the FAWT (*P* = 0.046), and DAWT or favorable histology WTs (FHWTs) were scattered across both clusters (Fig. 1d). Subsequent unsupervised clustering using 24 microenvironment cells led to the identification of two distinct subtypes: immune-rich and immune-cold. The immune-rich subtype had significantly higher infiltration of immune and stromal cells (both, *P* < 0.001). As expected, clear cell renal cell carcinoma (ccRCC) fell into the immune-rich subtype due to its high immune and stromal cell infiltration, whereas chromophobe renal cell carcinoma (ChRCC) demonstrated a cold infiltration pattern (Fig. 1e). Of note, all FAWT cases were found in the immune-rich subtype, while DAWT and FHWT were distributed across both clusters (Fig. 1e).

### Association between desert-like anaplastic WT, TP53 mutation and poor outcome

**Fig. 2:**
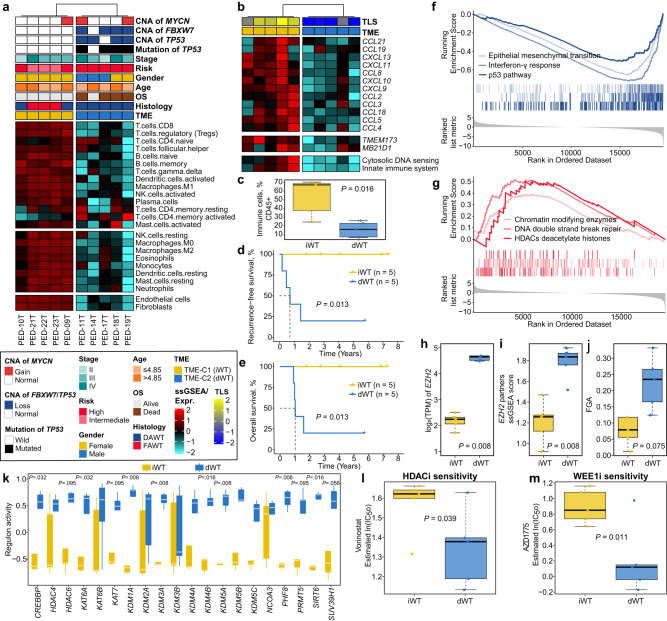
Association between desert-like Wilms tumors (WTs) and *TP53* mutation in diffuse anaplastic WT. **a** Heatmap showing two tumor microenvironment (TME) phenotypes using curated signatures of 24 microenvironment cell types. Clinicopathological information and genetic alteration were annotated at the top panel. DAWT, diffuse anaplastic Wilms tumor; FAWT, focal anaplastic Wilms tumor; ssGSEA, single-sample gene set enrichment analysis. **b** Heatmap showing different expression patterns of genes of interest. **c** Boxplot showing the distribution of bulk immune cell proportions between iWT (*n* = 5) and dWT (*n* = 5) using deconvolution approach with two-sided Mann-Whitney test. **d** Kaplan-Meier curve of recurrence-free survival (RFS) rate with two-sided log-rank test between two TME phenotypes (*n* = 10). **e** Kaplan-Meier curve of overall survival (OS) rate with two-sided log-rank test between two TME phenotypes (*n* = 10). GSEA curves showing significantly **f** downregulated and **g** upregulated pathways in dWT versus iWT. Boxplot showing expression of **h** *EZH2*, **i** enrichment score of *EZH2* partners, **j** fraction of genome altered (FGA), **k** chromatin remodeling regulons activity using two-sided Mann-Whitney test, and estimated drug sensitivity of **l** HDAC inhibitor and **m** WEE1 inhibitor between dWT (*n* = 5) and iWT (*n* = 5) using one-sided Student’s t-test. For all boxplots, the center line represents the median, box hinges represent first and third quartiles and whiskers represent ± 1.5× interquartile range. Source data are provided as a Source Data file.

We then examined the TME landscape of WTs in our SFCE-WT cohort (*n* = 10). Unsupervised clustering was performed on 24 microenvironment cells and two TME phenotypes were identified (Fig. 2a). TME-C1 (*n* = 5) showed higher infiltration in all types of microenvironment cells, including immune, myeloid, and stromal cells compared to TME-C2 (*n* = 5). Conversely, TME-C2 showed a generally desert-like phenotype. Therefore, we designated these two subtypes as either “infiltrated-like” WT (iWT) or “desert-like” WT (dWT). Of note, the dWT was tightly associated with *TP53* mutations (four [80%] vs 0; *P* = 0.048) compared to iWT. Within the DAWT cases, the desert-like tumors also enriched in *TP53* loss of function (mutations and copy number loss) compared to infiltrated-like tumors (*P* = 0.048). Analysis of the tertiary lymphoid structures (TLS) signature revealed a universal immune-depleted pattern in dWT (*P* = 0.008; Fig. 2b). Further deconvolution revealed significant lower proportion of bulk immune cells in dWT compared to iWT (*P* = 0.016; Fig. 2c). Patients with dWT also showed significantly poor RFS and OS (both, *P* = 0.013; Fig. 2d, e) compared to those with iWT.

As a universal absence of adaptive immune infiltration in *TP53*-mutated dWT was observed, and regarding the potential relationship between the cGAS-STING pathway, *TP53* mutation, and immunity^19^, we assessed the expression of these genes in our WT subtypes. Accordingly, in regard to the differential gene expression between dWTs and iWTs, we noted downregulation of the *TMEM173* gene encoding STING (5.1-fold decrease, false discovery rate [FDR] <0.001) and the *MB21D1* gene encoding cGAS (3-fold decrease, *P* = 0.015, FDR = 0.089) with associated negative enrichment for pathways related to cytosolic DNA sensing and innate immunity in dWT (Fig. 2b). Using Hallmark and Reactome associated gene sets, we performed gene set enrichment analysis (GSEA) and found that p53 (normalized enrichment score [NES] = −2.00, FDR = 0.003), interferon-γ (IFN-γ) (NES = −2.52, FDR = 0.0001), and epithelial mesenchymal transition (EMT; NES = −2.58, FDR = 0.003) pathways were significantly downregulated in dWT as compared to iWT (Fig. 2f), while HDACs deacetylate histones (NES = 1.66, FDR = 0.046), DNA double-strand break repair (NES = 2.20, FDR = 0.004), and chromatin-modifying enzymes (NES = 1.76, FDR = 0.004) pathways were activated (Fig. 2g). Notably, polycomb targets were upregulated in dWT, which was consistent with overexpression of the *EZH2* polycomb gene or signature of *EZH2* partners (both, *P* = 0.008; Fig. 2h, i). Although no significant association between TMB and TME phenotypes was observed (*P* = 0.92), we found that dWT tended to harbor a higher fraction of genome altered (FGA) as compared to iWT (*P* = 0.075; Fig. 2j).

Given the activation of the HDAC gene set in dWT, we then analyzed the regulons of potential epigenetic regulators relevant to cancerous chromatin remodeling. Out of 20 chromatin remodeling regulons, eight showed significantly higher activity in dWT relative to iWT (all, *P* < 0.05; Fig. 2k). HDAC inhibitors, a class of small-molecular therapeutics, are now approved by the Food and Drug Administration (FDA) as anticancer agents. Also, clinical trials of several HDAC inhibitors for use as anti-cancer drugs are ongoing^25^. In this context, we investigated the potential therapeutic effect of the FDA-approved HDAC inhibitor vorinostat. We estimated the IC_50_ for vorinostat through a predictive model-based strategy and found that dWT had a higher likelihood of responding to vorinostat considering significantly lower IC_50_ values as compared to iWT (*P* = 0.039; Fig. 2l). Since DNA damage checkpoint kinases ATR and WEE1 are among key regulators of DNA damage/repair pathways^26^, we then estimated the IC_50_ for WEE1 (AZD1775) and ATR (AZD6738) inhibitors based on promising early clinical trial results in other cancer types^27–31^. We inferred that dWT was more sensitive to WEE1 inhibitor (AZD1775) as compared to iWT considering significantly lower IC_50_ values (*P* = 0.011; Fig. 2m), while no statistical difference was observed concerning ATR inhibitor (AZD6738).

### External validation using an independent WT cohort

We then decided to validate our finding in 36 eligible DAWTs retrieved from the Therapeutically Applicable Research to Generate Effective Treatments (TARGET) cohort. Likewise, an infiltrated-like (*n* = 24) and a desert-like (*n* = 12) WT phenotype were identified using the same unsupervised strategy (Supplementary Fig. 2a, b). Consistently, deconvolution revealed significantly lower proportion of immune cells in dWT compared to iWT (*P* = 0.0007; Supplementary Fig. 2c). We also found that patients with dWT showed an inferior overall prognosis as compared to those with iWT (*P* = 0.0004; Supplementary Fig. 2d). In addition, subclass mapping revealed that the iWT (*P* = 0.01, FDR = 0.040) and dWT (*P* = 0.036, FDR = 0.072) subtypes exhibit significant transcriptomic similarity between the SFCE-WT and TARGET-WT cohorts (Supplementary Fig. 2e). Additionally, in the SFCE-WT and TARGET-WT cohorts, we discerned 2286 and 1582 upregulated genes (FoldChange > 2, *P* < 0.05, FDR < 0.25), along with 1833 and 284 downregulated genes (FoldChange <0.5, *P* < 0.05, FDR < 0.25) respectively, when comparing dWT to iWT. To quantify the extent of similarity, we applied the representation factor (RF) to calculate the statistical significance of the overlap between these gene sets. This led to the identification of a common subset comprising 737 upregulated genes (RF = 3.4, *P* < 0.0001) and 54 downregulated genes (RF = 2.8, *P* < 0.0001) (Supplementary Fig. 2f). These findings reinforce the robustness and reproducibility of our research. Interestingly, even though no statistical association was observed between *TP53* mutation, 17q loss, and TME phenotypes of DAWTs from the TARGET-WT cohort (both, *P* > 0.4), we found that the p53 pathway (NES = −1.41, FDR = 0.049) was significantly downregulated in dWT as well as IFN-γ (NES = −1.83, FDR = 0.003) and EMT pathways (NES = −1.81, FDR = 0.003; Supplementary Fig. 2g). Consistently, we noted a downregulation of the *TMEM173* (2.1-fold decrease, FDR < 0.001; Supplementary Fig. 2b) with no significant decrease identified for the *MB21D1* (1.3-fold decrease, *P* = 0.24, FDR = 0.42) with associated negative enrichment for pathways related to cytosolic DNA sensing and innate immunity in dWT. We observed that dWT showed significant upregulated HDACs deacetylate histones (NES = 1.56, *P* = 0.022, FDR = 0.180), DNA double-strand break repair (NES = 1.26, *P* = 0.035, FDR = 0.246), and chromatin-modifying enzymes (NES = 1.64, *P* = 0.013, FDR = 0.132) pathways (Supplementary Fig. 2h). We also observed overexpression of the *EZH2* polycomb gene and the signature of *EZH2* partners (both, *P* < 0.05; Supplementary Fig. 2i, j) in dWT as compared to iWT; no statistical difference was observed concerning FGA. In addition, 20 chromatin remodeling regulons were identified, of which 19 regulons showed significantly higher activity in dWT versus iWT (all, *P* < 0.05; Supplementary Fig. 2k). Consistently, dWT also showed a higher likelihood of responding to HDAC inhibitor vorinostat (*P* = 0.0099; Supplementary Fig. 2l) and WEE1 inhibitor AZD1775 (*P* = 0.046; Supplementary Fig. 2m), while no statistical difference was observed for ATR inhibitor AZD6738. Of note, we also identified two TME phenotypes using 60 eligible FHWTs from the TARGET-WT cohort (Supplementary Fig. 3a) where no prognostication-relevance was observed (*P* = 0.72; Supplementary Fig. 3b), suggesting the prognostic value of TME phenotypes in WT might be limited to tumors with anaplastic features.

### Prognostic value of CD8 and CD3 in anaplastic WTs treated with preoperative chemotherapy

**Fig. 3:**
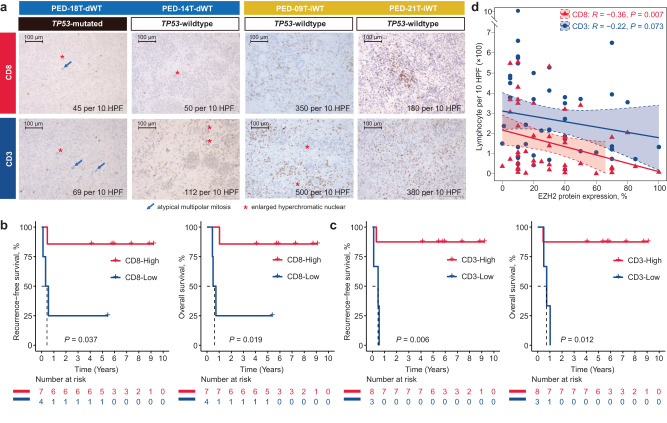
Prognostic value of tumor-infiltrating lymphocytes in pretreated anaplastic Wilms tumors (WTs). **a** Immunohistochemistry images of CD8 and CD3 in different tumor microenvironment (TME) subtypes of WT, including two samples for iWT (PED-18T and PED-14T) and two samples for dWT (PED-09T and PED-21T). Immunostaining using anti-CD3 and anti-CD8 antibodies was performed using standard protocol. Results on these tumors are shown at magnification x200 (scale bar 100 μm), with marked hallmark of anaplasia. Positive CD3 and CD8 lymphocytes were counted on 10 field at high-power fields (HPF, x400). **b** Kaplan-Meier curves of recurrence-free survival (RFS) (left panel) and overall survival (OS) (right panel) with two-sided log-rank test regarding the count of CD8 in anaplastic WTs (*n* = 11). **c** Kaplan-Meier curves of RFS (left panel) and OS (right panel) with two-sided log-rank test regarding the count of CD3 in anaplastic WTs (*n* = 11). **d** Scatter plot showing one-sided Spearman’s correlation between the percentage of EZH2 protein expression and count of CD3+ and CD8+ cells. Data are presented as linear model fits, with shaded areas representing the 95% confidence interval. Source data are provided as a Source Data file.

As the global downregulation of immune genes in dWTs, we investigated through immunohistochemistry (IHC) whether this could be related to the total of number of tumor-infiltrating lymphocytes (TILs) and immune checkpoint protein expression (PD-1 and PD-L1). Therefore, we counted TILs per 10 high-power fields (HPF) by assessing CD8 and CD3 for 11 samples, including 10 DAWTs and one FAWT, with available pathological slice images in our 21 anaplastic WTs. Specifically, two dWTs (PED-18T and PED-14T) and two iWTs (PED-21T and PED-09T) were investigated out of the 10 cases with transcriptome data. We observed over 5-fold higher CD8 (mean: 265 vs 47), as well as almost 5-fold higher CD3 (mean: 440 vs 90) in iWTs compared to dWTs (Fig. 3a, Supplementary Data 1). In addition, we found that both CD8 (*P* = 0.019) and CD3 (*P* = 0.012) were capable of stratifying the prognosis of anaplastic WTs regarding both RFS and OS (Fig. 3b, c). All patients with low CD8 or CD3 harbored *TP53* mutation while two cases had TP53 mutation in patients with high CD8 (100% vs 28.6%, *P* = 0.045), and three cases had TP53 mutation in patients with high CD3 (100% vs 37.5%, *P* = 0.012). We then questioned if CD8 and CD3 held prognostic value for non-anaplastic WTs. To this end, we collected another unselected cohort that contains 42 pretreated non-anaplastic WTs included in the French SIOP-2001 protocol, including three epithelial (7%), five blastemal (12%), seven stromal (17%), eight regressive (19%), and 19 mixed WTs (45%); these cases have at least one available slice to evaluate CD8 or CD3. Conversely, no statistical association was observed between the TILs count and prognosis in these cases (both, *P* > 0.05; Supplementary Fig. 4a, b). *EZH2* overexpression is closely associated with immune suppression in various cancers, as it can downregulate antigen presentation, induce immune checkpoint molecules, and modulate immune cell function, collectively enabling cancer cells to evade immune surveillance and escape immune-mediated destruction^32–34^. Consistently, we found that the expression of EZH2 protein showed strongly inverse correlation between CD8 (*R* = −0.36, *P* = 0.007) and CD3 (*R* = −0.22, *P* = 0.073) in WTs (Fig. 3d). In addition, out of the 53 pretreated WTs, membranous staining of PD-1 and PD-L1 was observed in one (2%) and four (8%) cases, respectively.

### Oncogenic signaling pathways identify WT cell lines with anaplasia

**Fig. 4:**
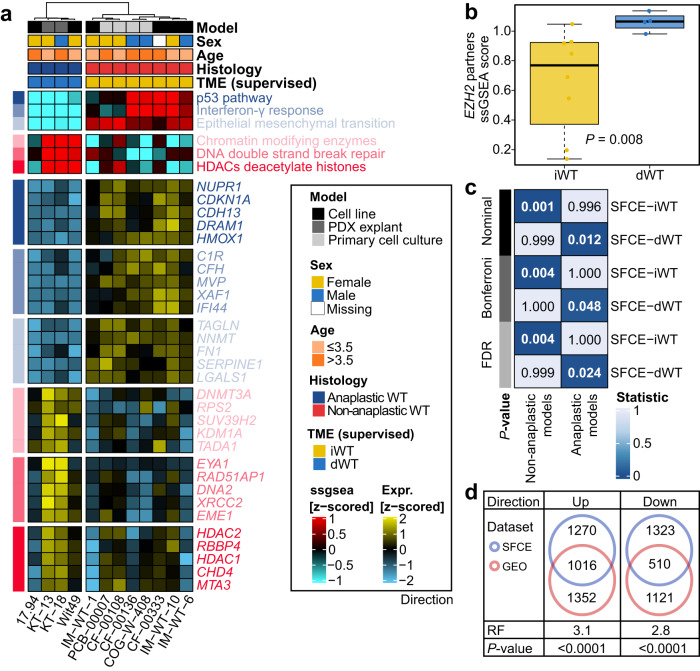
Validation using Wilms tumor (WT) cell lines and primary cultures. **a** Heatmap showing enrichment scores of oncogenic pathways in 12 WT cells/patient-derived xenograft (PDX)/primary-cultures. **b** Boxplot showing enrichment score of *EZH2* partners between iWT (*n* = 8) and dWT (*n* = 4) in GEO-WT dataset using two-sided Mann-Whitney test; the center line represents the median, box hinges represent first and third quartiles and whiskers represent ± 1.5× interquartile range. **c** Transcriptomic similarity between SFCE-WT subtypes and WT cells/PDX/primary-cultures histology using subclass mapping. Bonferroni and Benjamini-Hochberg correction (false discovery rate, [FDR]) methods were used to adjust *P*-values. **d** Venn diagrams with representation factor (RF) revealing significant overlapping of the differentially expressed genes in dWT versus iWT subtypes between SFCE-WT and GEO-WT datasets. Source data are provided as a Source Data file.

The development of an oncogenic state linked with the deregulation of cell signaling pathways is key to controlling cell growth and cell fate. To analyze whether the proposed WT phenotypes are reproducible based on the relevant oncogenic signaling pathways in Wilms cell lines, we collected 12 models from Gene Expression Omnibus (GEO), including six WT cell lines, four WT primary cultures, and two WT patient-derived xenograft (PDX) explants (WT cells/PDX/primary-cultures). We further conducted supervised clustering using the TME-specific cancer-related pathways on these models. Strikingly, anaplastic WT (*n* = 4) and non-anaplastic WT (*n* = 8) models were completely separated. Non-anaplastic WT models showed similar signatures to iWT; conversely, anaplastic WT models shared similar signatures with dWT (*P* = 0.002; Fig. 4a). Infiltrated-like models exhibited activation of p53 (*P* = 0.004), IFN-γ (*P* = 0.004), and EMT (*P* = 0.004) pathways, while desert-like models displayed activation of chromatin remodeling (*P* = 0.048), DNA repair (*P* = 0.079), and HDAC (*P* = 0.048) pathways. Although no statistical difference was observed in *EZH2* expression (*P* = 0.57), desert-like samples showed significantly higher enrichment of *EZH2* partners signature (*P* = 0.008; Fig. 4b). We then questioned if cell lines capture sufficient heterogeneity in tumors from the transcriptome aspect. To answer this, we performed subclass mapping and revealed that iWT (*P* = 0.001, FDR = 0.004) in the SFCE-WT cohort showed a significant overlap in transcriptome program with non-anaplastic models from the WT cells/PDX/primary-culture dataset, while dWT (*P* = 0.012, FDR = 0.048) showed significant similarity with those anaplastic models (Fig. 4c). In the GEO-WT dataset, we discovered 2368 upregulated and 1631 downregulated genes when comparing dWT to iWT. Furthermore, there was a common subset of 1016 upregulated genes (RF = 3.1, *P* < 0.0001) and 510 downregulated genes (RF = 2.8, *P* < 0.0001) shared with the SFCE-WT cohort (Fig. 4d). These findings suggested that including non-anaplastic WTs as controls allowed us to capture tumor heterogeneity to the maximum in anaplastic WT subtype. In addition, these data highly supported the conservation of the oncogenic pathway deregulation between patients’ samples and cancer cells and suggested that cancer cells could be used to test the sensitivity of therapeutic agents targeting components of a specific pathway.

### In vitro drug sensitivity test

**Fig. 5:**
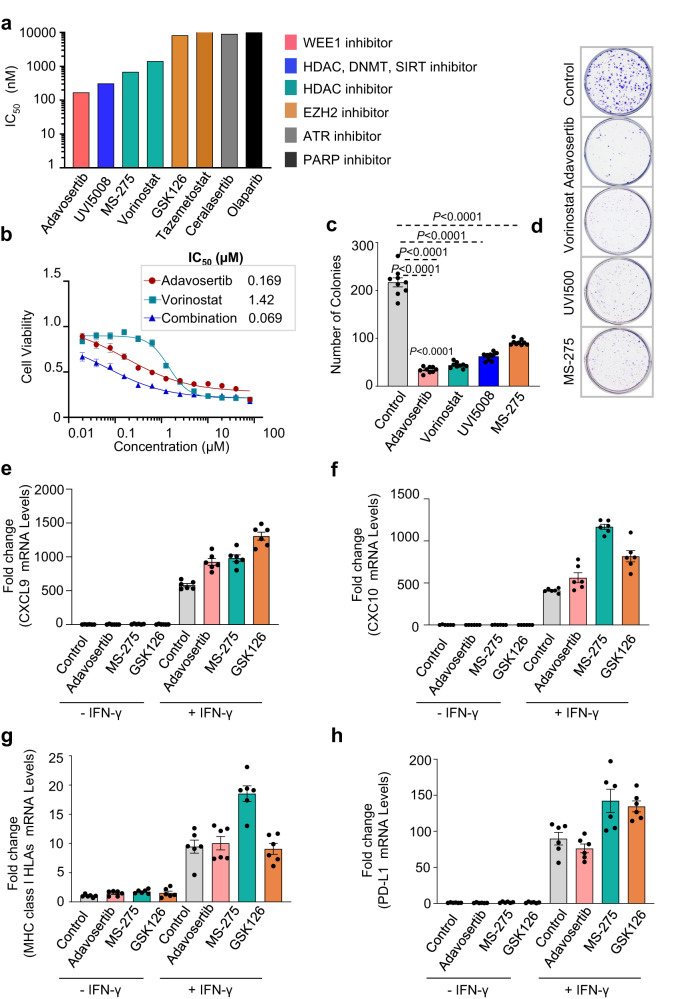
Testing of various epigenetic drugs. **a** Assessment of IC_50_ for Wilms tumor cell line (17.94) when exposed to different epigenetic drugs at a range of concentration for 72 h. The bar plot demonstrates ranked IC_50_ (nM) for different inhibitors in the 17.94 cell line (*n* = 4 independent experiments). **b** Dose-response effects of drugs combined treatment on the viability of the 17.94 cell line (*n* = 4 independent experiments). Clonogenic survival assay results depicted as **c** dot bar plot and **d** representative images showing the number of colonies in 17.94 cell line treated or untreated with various inhibitors (*n* = 9 biological samples, each with 3 technical replicates). Data was presented as mean ± standard deviation, and a two-sided Mann-Whitney test was applied to measure the *P* values between the control cells (DMSO) and the cells treated with various inhibitors. RT-qPCR was preformed to measure the mRNA levels for **e**, **f** chemokines (CXCL9 and CXCL10); **g** MHC class I surface receptor expression and **h** PD-L1 expression in 17.94 WT cells either stimulated or left unstimulated with IFN-γ in the absence/presence of various inhibitors (*n* = 2 independent experiments, with triplicates). Data was presented as mean ± standard deviation. Source data are provided as a Source Data file.

Regarding the conservation of the oncogenic state between human and cell lines, we tested the sensitivity of 17.94 WT cell lines to several drugs, including a WEE1 inhibitor (adavosertib), two HDAC inhibitors (MS-275 and vorinostat), a triple inhibitor (UVI500) targeting HDAC, DNMT and SIRT, two EZH2 inhibitors (GSK126 and tazemetostat), an ATR inhibitor (ceralasertib), and a PARP inhibitor (olaparib), based on in-silico analyses that suggested their potential efficacy in treating WTs. (Fig. 5a). We found that the 17.94 cells were most sensitive to the WEE1 inhibitor (adavosertib at 169 nM), followed by the triple inhibitor (UVI500 at 312.6 nM) and HDAC inhibitors (MS-275 at 684.4 nM and vorinostat at 1420 nM). The 17.94 cells were not sensitive to the EZH2, ATR, or PARP inhibitors, with IC_50_ values above 15 µM.

Since the WEE1 and HDAC inhibitors showed therapeutic efficacy, we hypothesized that combination therapy might increase their sensitivity in 17.94 WT cells. We tested this by using adavosertib and vorinostat alone and in combination over a range of concentrations, and found a synergistic effect of the combination (combinational index [CI] value = 0.069) (Fig. 5b). We also conducted a clonogenic survival assay to evaluate the effectiveness of each inhibitor individually and observed a significant decrease in 17.94 WT cells clonogenic potential when treated with adavosertib, UVI500, MS-275, or vorinostat, as shown by a reduction in the number of colonies compared to control cells (Fig. 5c, d).

Recent studies have shown that HDAC and WEE1 inhibition can have an immunostimulatory effect through various mechanisms, including activation of ERVs^35–39^. Therefore, we analyzed the effect of adavosertib on the expression of chemokines (CXCL9 and CXCL10) and PD-L1, as well as the promotion of antigen presentation in cells treated with IFN-γ relative to the control (i.e., Dimethyl sulfoxide [DMSO]). We found that 17.94 WT cells stimulated with IFN-γ and treated with either WEE1 (adavosertib) (2^−ΔΔCt^: CXCL9 = 924.4; CXCL10 = 561.52) or HDAC inhibitors (MS-275) (2^−ΔΔCt^: CXCL9 = 980.77; CXCL10 = 1,167.86) showed induction of CXCL9 and CXCL10 expression compared to the control (2^−ΔΔCt^: CXCL9 = 582.03; CXCL10 = 410.45) (Fig. 5e, f). The level of increase was similar to that observed with the EZH2 inhibitor (GSK126) (2^−ΔΔCt^: CXCL9 = 1,306.58; CXCL10 = 817.21), which has been shown to activate interferon-stimulated genes in different models (Fig. 5e, f). While the HDAC inhibitor MS-275 increased MHC class I HLAs (A-C and E-G) (2^−ΔΔCt^: 18.52) and PD-L1 expression (2^−ΔΔCt^: 142.36) in IFN-γ stimulated cells as compared to control (2^−ΔΔCt^: class I HLAs = 9.46; PD-L1 = 89.84), adavosertib (2^−ΔΔCt^: class I HLAs =10.04; PD-L1 = 76.31) had no effect. This suggests differences in action mechanisms between the two drugs (Fig. 5g, h). This is consistent with the EZH2 inhibitor (GSK126), which further enhanced PD-L1 level (2^−ΔΔCt^:134.62) after stimulation with IFN-γ in GSK126-treated cells compared to control cells (DMSO) (2^−ΔΔCt^: PD-L1 = 89.84) (Fig. 5h). These data suggest that blocking HDAC or tyrosine kinase cell-cycle progression regulator WEE1 may provide therapeutic vulnerabilities through cytotoxic effects and might also restore tumor immunogenicity, increasing the effects of immune checkpoint inhibitors in desert-like WTs.

## Discussion

WTs with anaplastic features are a rare and aggressive subset, accounting for only 10% of cases, and pose significant challenges in terms of treatment and cure^40^. Understanding the implications of these aggressive tumors for prognosis and therapeutic vulnerability is of utmost importance to guide the development of effective clinical trials for children affected by this disease. We reported herein the discovery and the validation of an aggressive subtype of DAWT, known as desert-like DAWT, which was characterized by depletion of immune and stromal cells, *TP53* alterations, chromosome instability, unique endogenous retroviruses expression, and activation of specific oncogenic signaling pathways. This subtype had a lower level of CD8 cytotoxic lymphocytes, suggesting immune evasion and poor outcomes. In contrast, we also identified a highly infiltrated DAWT subtype with high levels of cytotoxic lymphocytes and a signature of TLS. This suggests the presence of functional CD8+ cells and good outcomes, similar to what has been observed in different adults tumors, such as breast and nonsmall cell lung cancer^41^. High enrichment of the TLS signature also indicates its importance in adaptative immune response^42^.

Through multiple lines of evidence, we identified a striking interplay between *TP53* alterations, immune infiltration, overexpression of H3K27 methyltransferase *EZH2*, and activation of several chromatin remodeling pathways. The transcriptome analysis was backed up with profiling of the count of T cells, CD3 and CD8, which confirmed the loss of CD8+ cytotoxic cells in *TP53* mutated tumors, along with increased expression of *EZH2*. Our findings were further validated in the TARGET cohort, although patients in this cohort had not received pretreatment with chemotherapy, unlike the patients in the SFCE-SIOP cohort. Notably, although no *TP53* somatic mutations were enriched in desert-like DAWT in the TARGET cohort, the *TP53* pathway was downregulated, and this was associated with a downregulation of cGAS-STING, which senses cytosolic double-stranded DNA and triggers an antiviral innate immune response.

These findings suggest that mechanisms of immune evasion may differ between pretreated and untreated DAWT. In the pretreated tumors, mutant p53 might exert cancer-promoting gain-of-function activities by disrupting the cytoplasmic DNA sensing machinery and the cGAS-STING pathway, leading to the suppression of the innate immune response through altered cytokine production^19^. In untreated tumors, dysfunction of the p53 pathway may downregulate the cGAS-STING pathway and impair the activity and recruitment of T and myeloid cells, resulting in immune evasion^43^. Unlike adult cancers, WTs had low expression of the PD-L1 immune checkpoint. These results would explain the limited effectiveness of PD-1 inhibitors in this context, although definitive data using larger cohorts are expected to be reported soon^23^.

While the composition of the iWT and dWT subtypes within the DAWT may include samples from other histopathological subtypes (i.e., FAWT), our IHC analysis suggested that the TME plays a substantial role in determining the aggressiveness of DAWT tumors regardless of their initial classification. Specifically, CD8+ and CD3 + T cells are potent prognostic markers in pretreated anaplastic WTs. To better understand the characteristics and behavior of these immune cells in these aggressive tumors, further research is needed. Single-cell RNA sequencing (RNA-seq) analysis could also provide insights into the identity of these cells and how they interact with cancer cells through ligand-receptor interactions. This information could inform the development of targeted immunotherapies for WTs.

Our consistent finding of a tight association between *EZH2* overexpression and immune depletion in DAWT provide preliminary evidence suggesting that *EZH2* dysregulation contribute to shaping TME in these aggressive tumors. In several cancers overexpression of *EZH2* leads to the silencing genes associated with antigen presentation or tumor-suppressor genes^32^. Specifically, *EZH2* plays a crucial role in maintaining the low immunogenicity of bladder cancer cells by transcriptionally repressing cytokines and MHC class II antigen presentation genes, which leads to immune evasion and further shapes an immune-cold tumor microenvironment^44^. Cumulative evidence suggests that *EZH2* was instrumental in conferring tumor cells with resistance to immune-mediated destruction, thereby underscoring the merit of prioritizing *EZH2* as a target in immunotherapeutic strategies to potentially enhance treatment efficacy^34^.

Therapeutic options for aggressive tumors remain a challenge due to the lack of available immunocompetent murine models. Herein, we unraveled the conservation of oncogenic pathways in aggressive tumors in cell lines, suggesting the possibility to use those as a model to assess therapeutic options. Therefore, the use of 17.94 anaplastic WT lines—one of the rare models commercially available—showed the efficacy of WEE1 and HDAC inhibitors and HDAC in this setting. Notably, we also observed that WEE1 inhibition led to an increase in interferon-response genes following interferon-gamma activation; we could not exclude that this effect might be related to activating ERV as recently described, although this was not directly tested in our model^39^. A combination of WEE1 and HDAC inhibitors has been previously shown to be effective in human acute myeloid leukemia cells with various genetic mutations, regardless of *TP53* mutations, and this might be worth exploring as a treatment option in this population^45^.

We acknowledge the limitations of our in vitro work, which is based on a single cell line and does not fully capture the complexity of the tumor microenvironment. However, only one anaplastic cell (17.94) was commercially available, regarding the rarity of the disease. In addition, due to the unavailability of suitable murine cell lines or animal models for this specific tumor, conducting in vivo studies was not feasible to directly address the specific hypotheses of our study. To mitigate these limitations, we have leveraged multiple external datasets, including various models, to validate and strengthen our findings. Future studies should aim to address these limitations using larger cohorts allowing to better expand upon our findings.

In summary, we reported on the discovery of desert-like WT with prognostic and therapeutic relevance. We also provided mechanistic data on the efficacy of epigenetic therapies using HDAC with/without WEE1 inhibitors in this setting. Therefore, blocking either HDAC and/or tyrosine kinase cell-cycle progression regulator WEE1 may represent therapeutic vulnerabilities in these tumors, potentially restoring their tumor immunogenicity.

## Methods

### Patients and tumors selection

Clinicopathological data of WTs treated in France through the SIOP-2001 protocol were collected from children registered in 31 centers of the Société Française de Cancérologie de l’Enfant (SFCE); available frozen sample or formol-fixed-paraffin-embedded (FFPE) tissue blocks for tumor and normal kidney were included in the study. All tumors were evaluated by the local pathologist and reviewed at the national level by A.L.C. and L.D. DNA and RNA extractions were performed according to a standard protocol using the phenol-chloroform technique. Informed consent was obtained from all participating families to conduct the study. The study has been approved by the ethical committee of the Pitié-Salpêtrière Hospital (IDF-6, Ile de France) and conducted in accordance with the Helsinki Declaration. FFPE tissue blocks were used for IHC. Out of a total number of 180 WT, 37 (20.6%) cases were classified as high-risk, 141 (78.3%) as intermediate risk, and 2 (1.1%) as low-risk. Among those, 21 (11.6%) harbored focal (*n* = 4) or diffuse anaplasia (*n* = 17); those cases were subsequently used in this study for extensive genetic and/or transcriptomic analysis. Out of the 21 WT with anaplasia, 9 DAWT and 3 FAWT had matched normal kidney samples available. DNA has been extracted from all samples using DNeasy Blood & Tissue Kit (Quiagen) from 10–30 mg of tissues according to manufacturer’s instructions. For samples with available frozen tissues following DNA extraction, RNA has been extracted from additional 10–30 mg of tissues using the RNeasy Kit (QiagenQIAGEN) according to the manufacturer’s instructions. Quality control (QC) of extracted nucleic acids was performed using an Agilent 2100 Bioanalyzer. Twelve samples with matched germline and tumor DNA passed the QC and were subjected to WES. The remaining nine samples have either low quantity or quality allowing to perform WES, and were therefore used for targeted sequencing. RNA has been successfully extracted from 15 samples but only 10 samples passed Agilent QC (RNA integrity number > 7) and were therefore used for subsequent RNA-seq profiling. Clinicopathological details of those cases are reported in Supplementary Data 1. Eleven cases that have FFPE blocks are available for immune markers analysis including CD8 and CD3 counts. Follow-up and clinical data were collected in the SIOP 2001 database. The median follow-up time for the study cohort was 70 months (interquartile range 34–87 months).

### WTs treated with preoperative chemotherapy

A total of 53 pretreated WTs (11 anaplastic and 42 non-anaplastic) included in the French SIOP-2001 protocol were used to investigate the distribution and the prognostic role of TILs in WTs. Specifically, TIL’s (CD8 and CD3) architecture and counts, as well as immune checkpoint proteins (PD-1 and PD-L1) expression were analyzed.

### RNA sequencing

The total RNA for 10 cases (Supplementary Data 1) was converted into a library of template molecules for sequencing on the Illumina HiSeq 2000 according to the NuGen Ovation RNA-seq System V2 protocol. In brief, first, single-stranded cDNA was synthesized from 100 ng of DNase1-treated total RNA using a mix of DNA/RNA chimeric primers that hybridize to both the 50 portions of the poly (A) sequence and randomly across the transcript. Second, strand synthesis produced double-stranded cDNA, which was amplified using single-primer isothermal strand-displacement amplification. The resultant cDNA was fragmented to 200 bp (mean fragment size) with the S220 Focused-ultrasonicator (Covaris) and used to make barcoded sequencing libraries on the SPRI-TE Nucleic Acid Extractor (Beckman-Coulter). Libraries were quantitated by qPCR (KAPA Systems), multiplexed and sequenced—four samples per lane—on the HiSeq2000 using 75 bp paired-end sequencing. The resulting data were analyzed with the current Illumina bcl2fastq2 pipeline (v2.20) to generate raw FASTQ files. The raw, paired-end reads were aligned to the human reference genome, GRCh38/hg38, using MOSAIK (v1.1.0021) alignment software. MOSAIK works with paired-end reads from Illumina HiSeq 2000 and uses both a hashing scheme and the Smith-Waterman algorithm to produce gapped optimal alignments and to map exon junction-spanning reads with a local alignment option for RNA-seq. The resulting alignments were then saved as a standard bam file.

We then counted the mRNA-mapped reads annotated in GENCODE25 to generate the raw counts for each gene using the HTSeq-count script distributed with the HTSeq package. As a comparator, randomly selected 15 DAWTs and 15 FHWTs derived from previously published RNA-seq data^46^, with available expression profiles (Supplementary Data 3). As an additional control, The Cancer Genome Atlas level-1 raw data was authorized to assess and a dataset comprised of ccRCC (*n* = 15 cases)^47^, papillary renal cell carcinoma (PRCC; *n* = 15 cases)^48^, and ChRCC (*n* = 15 cases)^49^ listed in Supplementary Data 3 was also randomly selected. Another 10 ccRCC samples were collected from Pitié-Salpêtrière Hospital (PSL), resulting in four kidney cancer types with a total of 95 samples. To ensure comparability, we analyzed the data using the same framework for sequencing alignment and quantification with the same genome annotation. For all samples with raw count data, the number of fragments per kilobase of non-overlapped exon per million fragments mapped was computed first and transferred into transcripts per kilobase million (TPM) values, which are more comparable between samples^50^. All TPM values went through log_2_ transformation and were combined as a single cohort. To mitigate potential batch effects introduced by different cohorts, we employed the ComBat algorithm, a function included in the R package sva (v3.46.0), using default parameters. ComBat is an empirical Bayes framework that estimates and removes batch-specific variability, thus allowing us to effectively correct for any potential batch effects introduced by different cohorts. We included the cohort as a covariate in our statistical models to further account for any variability associated with the different cohorts. Principal component analysis was further performed to confirm the removal of cohort-specific effects.

### Whole exome-sequencing and somatic mutation detection

WES was performed on genomic DNA derived from paired tumor-normal samples related to those 12 cases using Agilent human V5 (51 Mb) capture and the HiSeq2000 sequencing platform (Supplementary Data 1). Average coverage was ~100x for cancer samples and 50x for matched normal. After raw paired-end reads from WES were aligned/mapped to the human genome reference (hg38) and PCR duplicate reads were removed by MOSAIK aligner, we then analyzed the resulting alignments using the Bayesian model-based software GigaBayes/FreeBayes that enables the efficient analysis of billions of aligned short-read sequences. The program evaluates each aligned base and base quality value at each position to indicate putative single-nucleotide variations (SNVs) and short insertions/deletions (indels), and their corresponding SNV probability value (PSNV). Base quality values are converted to base probabilities corresponding to each of the four possible nucleotides. Using a Bayesian formulation, a PSNV (or indel probability value, as appropriate) is calculated as the likelihood that multiple different alleles are present between the reference genome sequence and the reads aligned at that position. If the probability value exceeds a prespecified threshold, the SNV or indel candidate is reported in the output. In this study, we used a PSNV cutoff value (0.9) to define a high-confidence SNV or short indel candidate^51,52^. We filtered out all known SNVs/indels in the University of California, Santa Cruz (UCSC) dbSNP 135 and 1000 Human Genome Project single nucleotide polymorphisms (SNP) databases and kept any mutations, which are in the COSMIC database, curated by the Wellcome Trust Sanger Institute. The variation classification for each mutation was annotated by ANNOVAR (v2020Jun08) (Supplementary Data 2)^53^. Furthermore, we evaluated *TP53* mutations status using Sanger sequencing in the additional 9 DAWT and FAWT for which WES was not performed^54^. All *TP53* somatic mutations detected by WES were also confirmed by Sanger sequencing.

### External WT cohorts for validation

For in-silico validation, two external WT cohorts were used. The first validation cohort was retrieved from the TARGET initiative (accession number phs000218/DS-PEDCR), containing 114 WT cases with poor outcomes and for which transcriptome expression profiles and clinical outcomes were available. The TARGET-WT cohort included 42 DAWTs and 72 FHWTs that relapsed. The transcriptome raw count data were converted to TPM values accordingly. Genetic alteration of the TARGET-WT cohort was downloaded from the cBioPortal (https://www.cbioportal.org/). The second validation cohort was downloaded from the GEO (accession number GSE156065) according to the literature^55^. The GEO-WT dataset encompasses a total of 12 models; four out of these 12 WT models presented with anaplastic features, while the remaining eight models had no anaplastic features. We extracted transcriptome expression data in TPM values and corresponding model information for the GEO-WT dataset.

### Unsupervised consensus clustering for kidney cancers pooled mRNA profile

According to the upper decile median absolute deviation, genes with high variation were identified for unsupervised consensus clustering. We then performed unsupervised hierarchical clustering with k = 5 as the number of clusters, also the number of kidney cancer types, with a distance measurement of 1-Pearson’s coefficient and Ward’s linkage for each run, and the final hierarchical clustering based on the consensus matrix. The consensus process randomly extracted 90% of features and samples for 500 perturbations.

### Estimation of immune/stromal cells and tumor purity in WTs

The presence of infiltrating immune/stromal cells and tumor purity in tumor tissue was estimated by R package “estimate” (v1.0.13)^56^. As there were cases with relatively high tumor purity in the TARGET-WT cohort, we reasoned that samples with tumor purity greater than 0.98 were not robust for estimating microenvironment cell abundance. As a result, 96 out of 114 cases with *TP53* mutation status were kept in this study (Supplementary Data 4).

### Calculation of microenvironment cell abundance

We modified two gene signatures, LM22^57^ and MCPcounter (v1.2.0)^58^, to construct our compendium^59^. As LM22 does not contain signatures related to fibroblasts and endothelial cells, extra 40 genes were added to account for these cells (32 genes for endothelial cells and 8 genes for fibroblasts) from MCPcounter to our compendium, which consisted of 364 genes representing 24 microenvironment cell types (Supplementary Data 5). We used gene set variation analysis on these gene sets to generate enrichment scores for each cell by using the R package GSVA (v1.46.0). The signature of TLS was retrieved from the literature^60^, and the corresponding enrichment score for TLS was presented as a geometric mean value. To describe the constituent pattern (i.e., relative cell proportions) of microenvironment cell subsets within clusters, tumor purity was used to adjust the enrichment scores of each microenvironment cell subset. The adjusted enrichment score was calculated as the enrichment score divided by (1 - tumor purity)^61^. Major cellular compartments for epithelial tumors were deconvolved using TR4, a signature matrix consisting of bulk immune cell (CD45+), epithelial (EPCAM+), endothelial (CD31+), and fibroblast (CD10+) populations^62^. To impute cell type proportions, CIBERSORTx was applied independently to both SFCE and TARGET cohorts with default parameters^63^.

### Copy number variation analysis

Recurrent focal somatic copy number alterations were detected and localized using GISTIC2.0 through the GenePattern platform (https://www.genepattern.org/) with the thresholds of copy number amplifications/deletions being equal to ±0.2 and *q*-value threshold being equal to 0.25 with a confidence level of 75%^64^. FGA, which represents the percentage of genome that has been affected by copy number gains or losses, was calculated for a sample of our WT cohort based on copy number segment data as follows:

1 $$R={copy\; number\; of\; segments}/2$$

2 $${FGA}={B}_{r}/B$$

It is the fraction of genome with log_2_(copy number) larger than 0.2 versus genome with copy number profiled where *B*_*r*_ denotes the number of bases in segments with $\left|{\log }_{2}R\right| > 0.2$ and *B* represents the number of bases in all segments^65^. FGA scores of the TARGET-WT cohort were directly retrieved from cBioPortal^66^.

### Differential expression and function enrichment analyses

Differential expression analysis based on raw count data was conducted by R package edgeR (v3.40.0) where the Benjamini-Hochberg method was implemented to adjust nominal *P* values (FDR) for multiple tests. For GSEA based on transcriptome expression data, we prepared a pre-ranked gene list according to the descending ordered log_2_FoldChange value derived from differential expression analysis; we then leveraged the R package clusterProfiler (v4.6.0) to determine functional enrichment based on the Molecular Signature Database (MSigDB)^67,68^. Additionally, *EZH2* partners retrieved from the term KAMMINGA_EZH2_TARGETS in MSigDB were identified from a stem cell gene network analysis^69^, where coregulated and positively correlated gene sets were identified as *EZH2* targets or partners. The corresponding enrichment score was calculated using a single-sample GSEA (ssGSEA) approach through the R package GSVA (v1.46.0)^70^.

### Transcriptomic similarity

We performed subclass mapping through the GenePattern platform to evaluate the transcriptomic similarity between subclasses from independent data sets^71^. Mapping result is represented as a subclass association matrix filled with *P*-values for each subclass association.

### Regulon analysis

We used the R package RTN (v2.22.0) to reconstruct transcriptional regulatory networks (regulons) including a total of 71 candidate regulators that were relevant to cancerous chromatin remodeling^72,73^. Specifically, mutual information analysis and Spearman rank-order correlation computed the possible associations between a regulator and all potential targets from the transcriptome expression profile, and permutation analysis was used to erase associations. The FDR threshold was set as 0.01 for the SFCE-WT cohort and 0.00001 for the TARGET-WT cohort because of different sample sizes. A bootstrapping strategy removed unstable associations through 1000 times of resampling with a consensus bootstrap greater than 95%. Data processing inequality filtering eliminated the weakest associations in the triangles of two regulators and common targets. Individual regulon activity was estimated using a two-sided GSEA.

### Drug sensitivity prediction

Expression profile and drug sensitivity data of human cancer cell lines (CCLs) were downloaded from the largest publicly available pharmacogenomics database: Genomics of Drug Sensitivity in Cancer (GDSC) (https://www.cancerrxgene.org/). The half maximal inhibitory concentration (IC_50_) was provided as a measure of drug sensitivity, and lower IC_50_ values indicate increased sensitivity to treatment. A total of 727 CCLs with both expression and drug sensitivity records were identified. *K*-nearest neighbor imputation was applied to impute the missing IC_50_ values. We then employed the R package pRRophetic (v0.5) to predict the chemotherapeutic sensitivity for each WT case with default settings^74^; the estimated IC_50_ of each sample treated with a specific chemotherapy drug was obtained by ridge regression, and prediction accuracy was measured through tenfold cross-validation with the GDSC training set.

### Immune infiltrate assessment

The immunohistochemical characterization of the immune response was performed using whole-slide staining. The FFPE samples were cut into 4 µm thick sections and placed on glue-coated glass slides for IHC. Sections were deparaffinized in xylene, rehydrated sequentially in ethanol, and placed into a phosphate-buffered saline solution (PBS; pH 7.4). The following antibodies were tested: CD3 and CD8. Details of antibodies are summarized in Supplementary Table 1. The latter immunoreactions were evaluated by two pathologists (L.D. and A.L.C.), who chose a minimum of 10 HPF (x400) representative of each case.

### IHC assessment of EZH2

To determine the global levels of EZH2 in tumors, we performed Tissue Microarrays (TMAs), including at least three replicate tumor samples of 4 mm spots taken from donor tissue blocks in a highly representative fashion. We analyzed the level of the EZH2 using the Abcam antibody (ab191080). The level of staining, referring to the percentage of positive cells showing nuclear positivity, was assessed independently by two pathologists (L.D. and A.L.C.), who were blinded to clinical and pathological data.

### Cell viability assays

WT 17.94 cell line was chosen as was one of the rare anaplastic Wilms cell lines commercially available^75^. The 17.94 cell line (cat no. ACC 741) was purchased from Leibniz-Institut DSMZ-Deutsche Sammlung von Mikroorganismen und Zellkulturen GmbH. The identity of the cell line was confirmed by short tandem repeat (STR) analysis. The 17.94 cells were tested negative for Mycoplasma and were maintained in media (DMEM [GIBCO, 41965-039], 10% FBS [PAN biotech, P30-3602], MEN-NEAA [Gibco, 11140-035], 1mMPyr-Na [Gibco, 11360-039], and Pen strep [Gibco, 15140-122]). The vorinostat (cat. no. HY-1022), entinostat (MS-275; cat. no. HY-12163), GSK126 (cat. no. HY-13470), tazemetostat (EPZ-6438; cat. no. HY-13803), adavosertib (cat. no. HY-10993), ceralasertib (cat. no. HY-19323), and Olaparib (cat. no. HY-10162) inhibitors were purchased from MedChemExpress. The UVI5008 (triple inhibitor) was given as a gift by Hinrich Gronemeyer (IGBMC). All the inhibitors were dissolved in DMSO for the study. The 17.94 cell line was trypsinized (Trypsin-EDTA [Gibco, 25200-072]), counted (5000 cells), and seeded in a 96-well plate on day 0. After 48 h incubations, the media was replaced with the 100 µl media with a required concentration of inhibitor or DMSO vehicle (as control sample) per well. To calibrate the IC50, different concentrations (~20 nM to 80 µM, on a logarithmic scale) for each inhibitor were applied to the 17.94 cell line. Each experiment was performed in quadruplicate (*n* = 4 independent experiments) and kept in an incubator for 72 h at 37 °C, supplemented with 5% CO_2_. The assay was stopped by adding PrestoBlue Cell Viability Reagent (cat. no. A13261, Invitrogen) to each well following the manufacture protocol. The output was read with a microplate reader (Berthold Mithras LB940). For statistical analysis, IC_50_ was obtained by fitting four-parameter logistic curves using the ratio obtained for each inhibitor absorbance value to the control using GraphPad Prism (version 9).

### Combinational effect of WEE1 inhibitor and vorinostat on WT cell line

The 17.94 cell lines were plated in 96-well plates according to the methodology mentioned in the Cell viability assay section. To calibrate IC_50_, a range of concentration (~20 nM to 80 µM, on a logarithmic scale) was selected. The cells were treated alone or in combination with the same concentrations of vorinostat and adavosertib. They were kept for 72 h in the incubator at 37 °C supplemented with 5% CO_2_. To end the assay, PrestoBlue cell viability reagent (cat. no. A13261, Invitrogen) was added according to manufactures instructions and the plates were read out using the microplate plate reader (Berthold Mithras LB940). Each experiment was performed in quadruplicate (*n* = 4 independent experiments); and the IC_50_ obtained from biological experiments was used to calculate the CI using the formula below:

3 $${{{{{\rm{CI}}}}}}=\frac{{D}_{1}}{{{Dx}}_{1}}+\frac{{D}_{2}}{{{Dx}}_{2}}$$

Here, *D* represents the combined concentration of the drugs, and *Dx* shows the concentration of single drug alone. The CI value < 1 indicates synergism, = 1 shows additivity, and > 1 represents antagonism, respectively^76–78^.

### Clonogenic survival assessment

WT 17.94 cells were seeded at 5000 cell density in six-well plates and kept at 37 °C supplemented with 5% CO_2_. After 48 h of incubation, the cells were treated with various inhibitors according to the IC_50_ obtained for each inhibitor. The cells were incubated for 48 h with the various inhibitors and later media was replaced with the fresh one. The cells were kept in the incubator for an additional 20 days by adding 1 ml of media after three days. At the end of the assay, the media from cells were removed, washed with cold PBS (1x), and fixed with 3.4% formaldehyde. The colonies were stained with crystal violet. Later pictures were taken and then the colonies were counted using the ImageJ (1.53n). Each biological experiment was performed in triplicate. The data is presented as mean ± standard deviation.

### Cell line testing with drugs with/without IFN-γ stimulation

WT 17.94 cells were randomly divided into four groups: 1) a control group that was not treated with an inhibitor and stimulated with the IFN-γ; 2) cells treated with the inhibitor using IC_50_ concentration (for 48 h); 3) cells stimulated with the IFN-γ (20 ng) (for 12 h); 4) cells treated with the inhibitor (for 48 h) and stimulated with the IFN-γ (20 ng) (for12 h). For each group, two biological experiments were performed in triplicates. The cell lysates were collected for RNA extraction using standard-based TRIzol RNA extraction according to manufacturer instructions. Reverse transcription-quantitative PCR (RT-qPCR) was performed using the SuperScript™ IV Reverse Transcriptase (Invitrogen, 18090050) using random hexamer primer (Invitrogen, SO142). The RT-qPCR was performed using a 20 μL reaction system in a real-time PCR machine with SYBR green master mix (LightCycler-480 SYBR, Roche) according to manufacture instructions. The relative expression of the genes *CXCL9* (F: TCAATTTTCTCGCAGGAAGG; R: ACCAACCAAGGGACTATCCAC), CXCL10 (F: CGTGGACAAAATTGGCTTG; R: GCTGTACCTGCATCAGCATTAG), PD-L1 (F: GGTTGTGGATCCAGTCACCT; D: TGTGCTGGTCACATTGAAAA), and HLA class I (covering *HLA*s *A-C* and *E-G*; (F: CCTACGACGGCAAGGATTAC; R: TGCCAGGTCAGTGTGATCTC) were normalized according to housekeeping gene Actin (F: ACATCTGCTGGAAGGTGGAC; R: CCCAGCACAATGAAGATCAA) by calculating the standard 2^−ΔΔCt^ method. Each biological experiment was performed in triplicate. The data is presented as mean ± standard deviation.

### Statistical analyses

All statistical tests were executed by R (v4.2.2). The Student’s t-test or Mann-Whitney test were used for analyzing continuous data, while Fisher’s exact test was used for analyzing categorical data. Spearman’s coefficient was used for evaluating correlations between two continuous variables. Survival rates were analyzed using Kaplan-Meier curves, with differences determined using a log-rank test through R package “survival” (v3.4.0). Relationships between lymphocyte count and patient survival were computed using R package survminer (v0.4.9) where patients were stratified using an optimal cutoff determined by the maximally selected rank statistics. Mutual exclusivity analysis was conducted using a one-side Fisher’s exact test. The statistical significance of the overlap between two groups of genes was estimated by calculating the RF and associated probability. The RF is calculated as the ratio of the number of overlapping genes to the expected number of overlapping genes based on the number of genes in each group (i.e., differentially expressed genes) and the total number of genes in the genome that had robust expression (raw count > 0 in all the samples) across different cohorts (16,475 genes annotated by GENCODE25). RFs of > 1, <1, and 1 respectively indicate more overlap than expected from two independent groups, less overlap than expected, and that the two groups have the number of genes expected for independent groups of genes. The probability of overlapping was estimated by exact hypergeometric probability or normal approximation when the exact hypergeometric probability was difficult to calculate. For unadjusted comparisons, a *P* value < 0.05 was considered statistically significant.

### Reporting summary

Further information on research design is available in the Nature Portfolio Reporting Summary linked to this article.

### Supplementary information

Supplementary Information

Description of Additional Supplementary Information

Supplementary Data 1

Supplementary Data 2

Supplementary Data 3

Supplementary Data 4

Supplementary Data 5

Reporting Summary

### Source data

Source Data

## Data Availability

The raw RNA-seq data of the French SIOP-2001 WT cohort have been deposited in the GEO database under accession code GSE224266. The variant call data of French SIOP-2001 WT study are provided in Supplementary Data 2; other data required to verify the published results are provided as Source Data. The underlying raw WES data are not available for this manuscript because the patients’ family did not consent to the release, sharing or distribution of the data. The RNA-seq data for TARGET-WT cohort under accession code phs000218/DS-PEDCR are available under controlled access [https://gdc.cancer.gov]^46^. The genetic alteration data for TARGET-WT cohort is publicly available in the cBioPortal database under project Pediatric Wilms’ Tumor (TARGET, 2018) [https://www.cbioportal.org/]^46^. The remaining publicly available data used in this study are available in the GEO database under accession code GSE156065^55^, in the ArrayExpress database under accession code E-MTAB-3610^79^, and in the GDSC database for drug screening data [https://www.cancerrxgene.org/]^80^. The remaining data are available within the Article, Supplementary Information or Source Data file. Source data are provided with this paper.

## Code availability

The central script and customized functions used in this study are available on the GitHub website at https://github.com/xlucpu/WTs/tree/main.
