# Supplementary material for: Delineating the interplay between oncogenic pathways and immunity in anaplastic Wilms tumors
Source: Nat Commun. 2023 Nov 30;14:7884. doi: 10.1038/s41467-023-43290-3 (PMC10689851; doi:10.1038/s41467-023-43290-3)
Supplement: Supplementary file 1 — Supplementary Information [file 41467_2023_43290_MOESM1_ESM.pdf]

Supplementary Information for

Delineating the interplay between oncogenic pathways and immunity in anaplastic Wilms tumors

Su *et al*.

SUPPLEMENTARY FIGURES

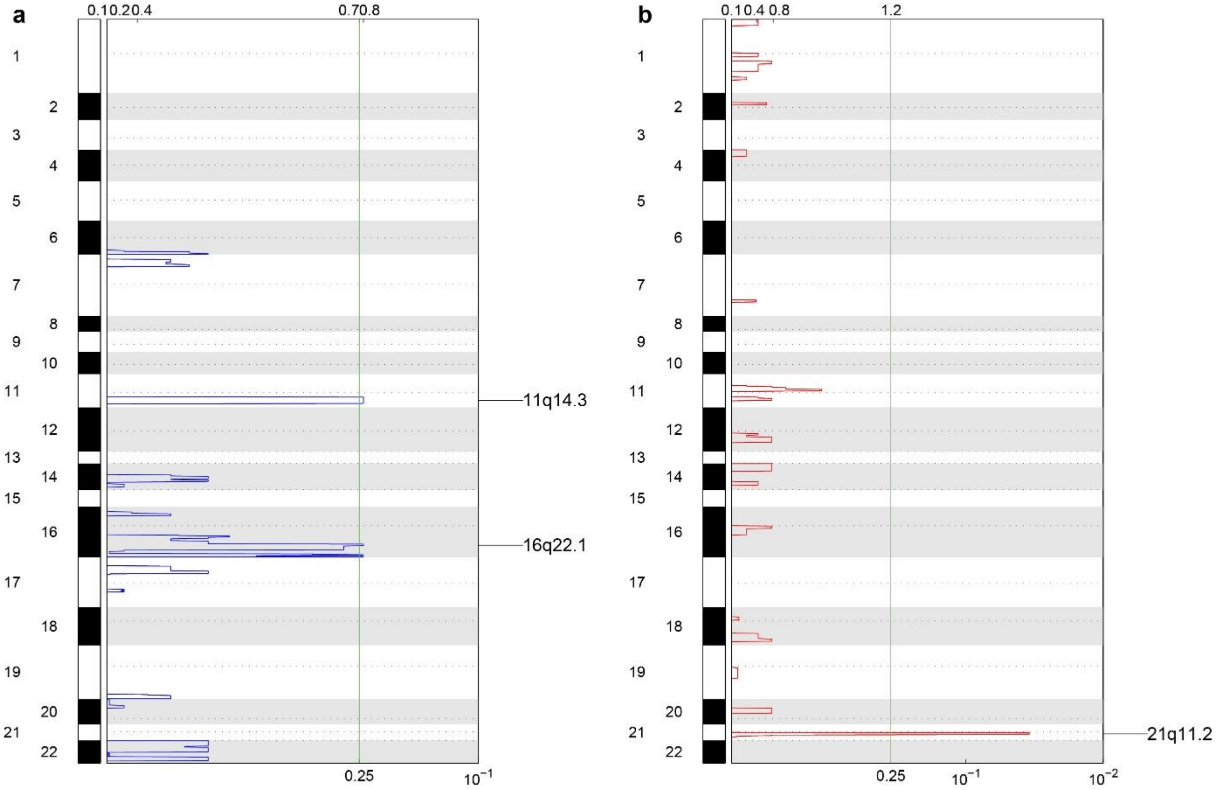

Supplementary Figure 1. Recurrent of copy number alteration (CNA). CNA identified by GISTIC2.0 for **a** copy number deletion and **b** copy number amplification. Source data are provided as a Source Data file.

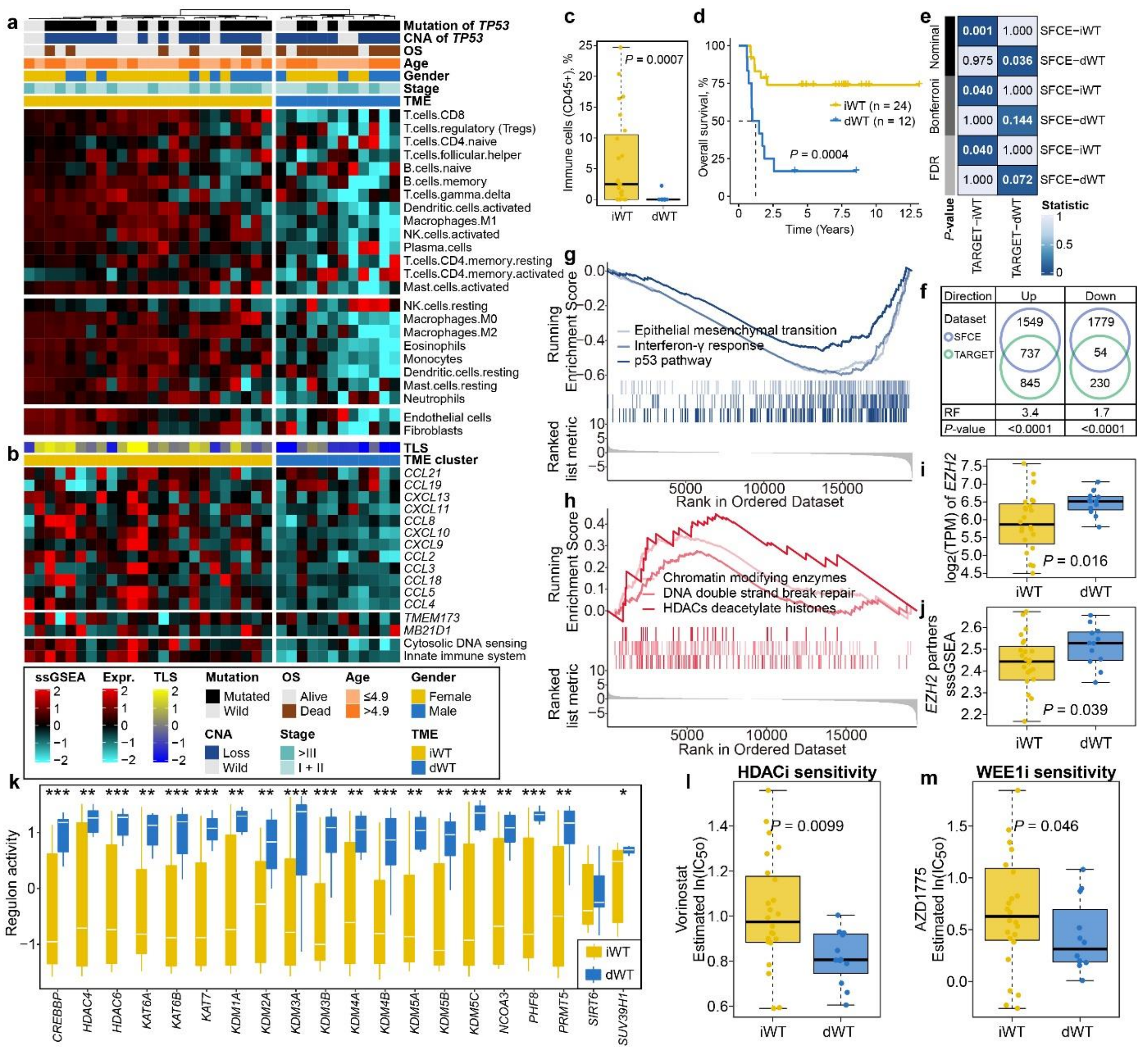

Supplementary Figure 2. Validation using diffuse anaplastic Wilms tumor (DAWT) samples from TARGET-WT cohort. a Heatmap showing two tumor microenvironment (TME) phenotypes using curated signatures of 24 microenvironment cell types. Clinicopathological information and genetic alteration were annotated in the top panel. **b** Heatmap showing different expression pattern of genes of interest. **c** Boxplot showing distribution of bulk immune cell proportions between iWT (n = 24) and dWT (n = 12) using deconvolution approach with two-sided Mann-Whitney test. **d** Kaplan-Meier curve of overall survival (OS) rate with two-sided log-rank test between two TME phenotypes. **e** Subclass mapping analysis revealing statistical similarity between SFCE and TARGET cohort regarding two TME phenotypes. Bonferroni and Benjamini-Hochberg correction (false discovery rate [FDR]) methods were used to adjust *P*-values. **f** Venn diagrams with representation factor (RF) revealing significant overlapping of the differentially expressed genes in dWT versus iWT subtypes between SFCE-WT and TARGET-WT cohorts. Gene set enrichment analysis curves showing significantly **g** downregulated and **h** upregulated pathways in dWT versus iWT. Boxplot showing expression of **i** *EZH2*, **j** enrichment score of *EZH2* partners, **k** chromatin remodeling regulons activity (* $P < 0.05$, ** $P < 0.01$, *** $P < 0.001$) using two-sided Mann-Whitney test, and estimated drug sensitivity of **l** HDAC inhibitor and **m** WEE1 inhibitor between two TME phenotypes using one-sided

Student's t-test. For all boxplots, the center line represents the median, box hinges represent first and third quartiles and whiskers represent ± 1.5× interquartile range. Source data are provided as a Source Data file.

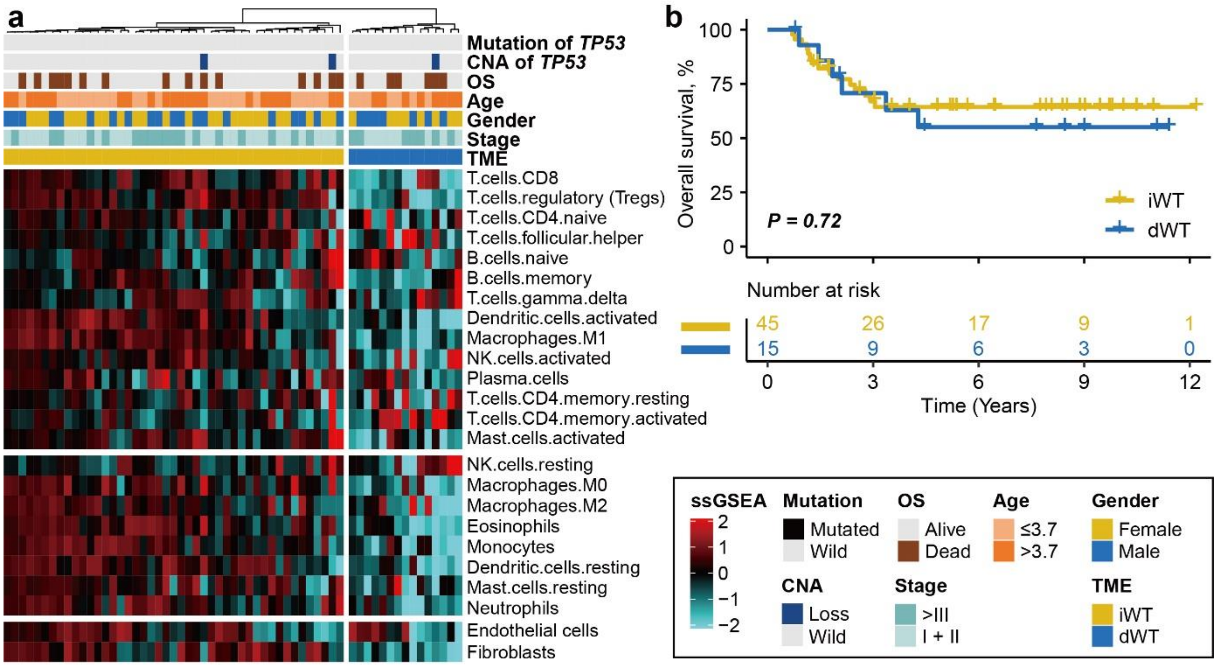

Supplementary Figure 3. Identification of tumor microenvironment (TME) phenotypes in favorable histology Wilms tumor (FHWT) from TARGET-WT cohort. a Heatmap showing two TME phenotypes (iWT, n = 45; dWT, n = 15) using curated signatures of 24 microenvironment cell types. Clinicopathological information and genetic alteration were annotated at the top panel. **b** Kaplan-Meier curve of overall survival (OS) rate with two-sided log-rank test between two TME phenotypes. Source data are provided as a Source Data file.

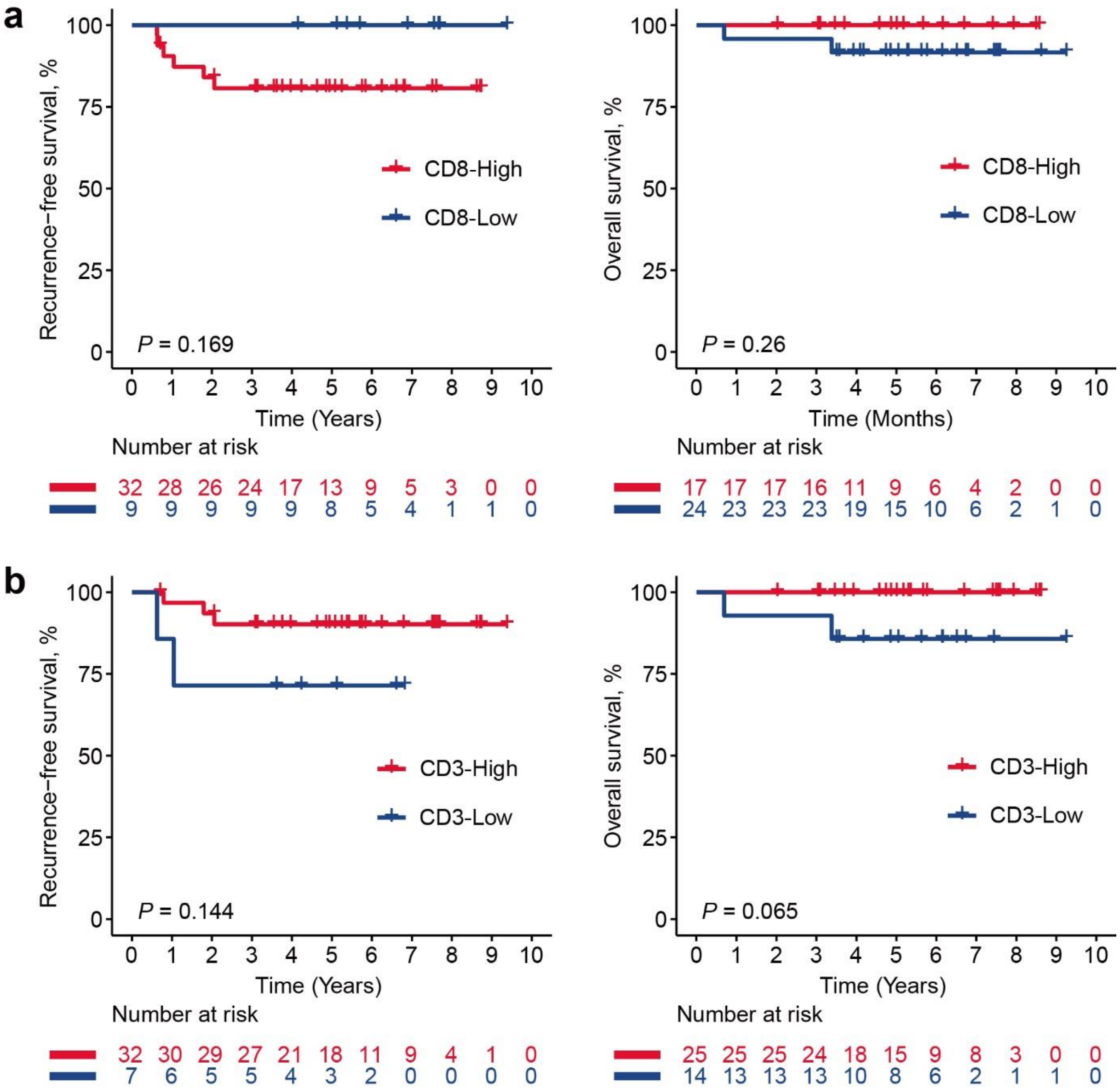

Supplementary Figure 4. Prognostic value of tumor-infiltrating lymphocytes in pretreated non-anaplastic WTs. a Kaplan-Meier curves of recurrence-free survival (RFS) (left panel) and overall survival (OS) (right panel) with two-sided log-rank test regarding the count of CD8 in non-anaplastic WTs (n = 41; one not available for CD8 count). **b** Kaplan-Meier curves of RFS (left panel) and OS (right panel) with two-sided log-rank test regarding the count of CD3 in non-anaplastic WTs (n = 39; three not available for CD3 count). Source data are provided as a Source Data file.

SUPPLEMENTARY TABLES

Supplementary Table 1. Immunohistochemical details

Antibody	Commercial source	Clone	Dilution	Incubation (min)	Demasking (PH)
CD3	DAKO	Polyclonal	RTU	32	8
CD8	DAKO	C8/144B	1/1000	36	8
EZH2	AbCAM	ab191080	1/500	36	8

Abbreviation: RTU, ready to use
