# Supplementary material for: Delineating the interplay between oncogenic pathways and immunity in anaplastic Wilms tumors
Source: Nat Commun. 2023 Nov 30;14:7884. doi: 10.1038/s41467-023-43290-3 (PMC10689851; doi:10.1038/s41467-023-43290-3)
Supplement: Supplementary file 2 — Description of Additional Supplementary Information [file 41467_2023_43290_MOESM2_ESM.pdf]

Description of Additional Supplementary Files

File Name: Supplementary Data 1
Description: Clinicopathological data of 21 Wilms tumors treated in France through the SIOP protocol 2001

File Name: Supplementary Data 2
Description: Somatic mutations called from whole-exome sequencing in 12 Wilms tumors

File Name: Supplementary Data 3
Description: Summarization of a mixed kidney cancer cohort with 95 randomly selected cases

File Name: Supplementary Data 4
Description: Tumor purity and TP53 alterations in TARGET-WT cohort

File Name: Supplementary Data 5
Description: Curated compendium of microenvironment genes related to 24 microenvironment cell subsets
