# Supplementary material for: Delineating the interplay between oncogenic pathways and immunity in anaplastic Wilms tumors
Source: Nat Commun. 2023 Nov 30;14:7884. doi: 10.1038/s41467-023-43290-3 (PMC10689851; doi:10.1038/s41467-023-43290-3)
Supplement: Supplementary file 8 — Reporting Summary [file 41467_2023_43290_MOESM8_ESM.pdf]

nature portfolio

Corresponding author(s): Gabriel G. Malouf

Last updated by author(s): Oct 16, 2023

Reporting Summary

Nature Portfolio wishes to improve the reproducibility of the work that we publish. This form provides structure for consistency and transparency in reporting. For further information on Nature Portfolio policies, see our Editorial Policies and the Editorial Policy Checklist.

Statistics

For all statistical analyses, confirm that the following items are present in the figure legend, table legend, main text, or Methods section.

n/a	Confirmed	
☐	☒	The exact sample size (*n*) for each experimental group/condition, given as a discrete number and unit of measurement
☐	☒	A statement on whether measurements were taken from distinct samples or whether the same sample was measured repeatedly
☐	☒	The statistical test(s) used AND whether they are one- or two-sided<br>*Only common tests should be described solely by name; describe more complex techniques in the Methods section.*
☐	☒	A description of all covariates tested
☐	☒	A description of any assumptions or corrections, such as tests of normality and adjustment for multiple comparisons
☐	☒	A full description of the statistical parameters including central tendency (e.g. means) or other basic estimates (e.g. regression coefficient) AND variation (e.g. standard deviation) or associated estimates of uncertainty (e.g. confidence intervals)
☐	☒	For null hypothesis testing, the test statistic (e.g. *F*, *t*, *r*) with confidence intervals, effect sizes, degrees of freedom and *P* value noted<br>*Give P values as exact values whenever suitable.*
☒	☐	For Bayesian analysis, information on the choice of priors and Markov chain Monte Carlo settings
☒	☐	For hierarchical and complex designs, identification of the appropriate level for tests and full reporting of outcomes
☐	☒	Estimates of effect sizes (e.g. Cohen's *d*, Pearson's *r*), indicating how they were calculated

Our web collection on statistics for biologists contains articles on many of the points above.

Software and code

Policy information about availability of computer code

Data collection	No software was used for data collection.
Data analysis	bcl2fastq2 (v2.20), MOSAIK (v1.1.0021), HTSeq-count, ANNOVAR, GigaBayes, FreeBayes, estimate (v1.0.13), MCPcounter (v1.2.0), CIBERSORTx online tool, GenePattern online platform, MOVICS (v0.99.17), ClassDiscovery (v3.4.0), edgeR (v3.40.0), clusterProfiler (v4.6.0), GSVA (v1.46.0), RTN (v2.22.0), pRRophetic (v0.5), GISTIC (v2.0), SubMap, survival (v3.4.0), survminer (v0.4.9). The central script and customized functions used in this study are available on the GitHub website at https://github.com/xlucpu/WTs/tree/main.

For manuscripts utilizing custom algorithms or software that are central to the research but not yet described in published literature, software must be made available to editors and reviewers. We strongly encourage code deposition in a community repository (e.g. GitHub). See the Nature Portfolio guidelines for submitting code & software for further information.

Data

Policy information about availability of data

All manuscripts must include a data availability statement. This statement should provide the following information, where applicable:

- Accession codes, unique identifiers, or web links for publicly available datasets
- A description of any restrictions on data availability
- For clinical datasets or third party data, please ensure that the statement adheres to our policy

The raw RNAseq of the French SIOP-2001 WT cohort have been deposited in the GEO database under accession code GSE224266 [https://www.ncbi.nlm.nih.gov/geo/query/acc.cgi?acc=GSE224266]. The variant call data of French SIOP-2001 WT study are provided in Supplementary Data 2; other data required to verify the

published results are provided as Source Data. The underlying raw sequencing data of whole-exome sequencing are not available for this manuscript. Family of patients were not consented for the release, sharing or distribution of the underlying whole-exome sequencing data. The RNA-seq data for TARGET-WT cohort under accession code phs000218/DS-PEDCR are available under controlled access [https://gdc.cancer.gov]46. The genetic alteration data for TARGET-WT cohort is publicly available in the cBioPortal database under project Pediatric Wilms' Tumor (TARGET, 2018) [https://www.cbioportal.org/]46. The remaining publicly available data used in this study are available in the GEO database under accession code GSE156065 [https://www.ncbi.nlm.nih.gov/geo/query/acc.cgi?acc=GSE156065]55, in the ArrayExpress database under accession code E-MTAB-3610 [https://www.ebi.ac.uk/biostudies/arrayexpress/studies/E-MTAB-3610]79, and in the GDSC database for drug screening data [https://www.cancerrxgene.org/]80. Source data are provided with this paper. The remaining data are available within the Article, Supplementary Information or Source Data file.

Research involving human participants, their data, or biological material

Policy information about studies with human participants or human data. See also policy information about sex, gender (identity/presentation), and sexual orientation and race, ethnicity and racism.

Reporting on sex and gender	Anaplastic Wilms tumors from 21 patients were analyzed including 12 females and 9 males. The findings in this study were not exclusive to either sex.
Reporting on race, ethnicity, or other socially relevant groupings	A total of 21 anaplastic WT patients were included in the study, with 17 having diffuse anaplastic WT and 4 having focal anaplastic WT. The cohort consisted of 12 females and 9 males, ranging in age from 2 to 14 years old. Among these patients, 11 were found to have TP53 somatic mutations. Furthermore, among the patients with localized disease at diagnosis, 2 were classified as stage I, 4 as stage II, and 10 as stage III. On the other hand, all 5 patients with metastatic disease were classified as stage IV.
Population characteristics	Patients in this study were diagnosed as Wilms tumor with anaplastic features. The cohort consisted of 12 females and 9 males, with a mean age of 5.4 years, ranging from 1.8 to 14.3 years. Among these patients, 11 were found to have TP53 somatic mutations. Furthermore, among the patients with localized disease at diagnosis, 2 were classified as stage I, 4 as stage II, and 10 as stage III. On the other hand, all 5 patients with metastatic disease were classified as stage IV.
Recruitment	Due to the rarity of the tumor we retrospectively analyzed the 21 WTs with anaplastic features and there was no prospective recruitment.
Ethics oversight	Informed consent was obtained from all families. The study has been approved by the ethical committee of the Pitié-Salpêtrière Hospital (IDF-6, Ile de France) and conducted in accordance with the Helsinki Declaration.

Note that full information on the approval of the study protocol must also be provided in the manuscript.

Field-specific reporting

Please select the one below that is the best fit for your research. If you are not sure, read the appropriate sections before making your selection.

[x] Life sciences [ ] Behavioural & social sciences [ ] Ecological, evolutionary & environmental sciences

For a reference copy of the document with all sections, see nature.com/documents/nr-reporting-summary-flat.pdf

Life sciences study design

All studies must disclose on these points even when the disclosure is negative.

Sample size	No a priori calculation of sample size was performed as analyses were conducted on the limited availability of access to this human tumor samples. It is important to note that anaplastic Wilms tumors are rare and aggressive, representing only 10% of all Wilms tumors. Despite the challenges posed by the rarity of these tumors, we were able to utilize samples from the French SIOP-2001 trial, which provided us with valuable clinical data of good quality.
Data exclusions	Out of a total number of 180 WT, 37 (20.6%) cases were classified as high-risk, 141 (78.3%) as intermediate risk, and 2 (1.1%) as low-risk. Among those, 21 (11.6%) harbored focal (n = 4) or diffuse anaplasia (n = 17); those 21 anaplastic WT cases were subsequently used in this study for extensive genetic and/or transcriptomic analysis.
Replication	The experiments were performed independently with range varying between 2-9 as specified in the manuscript.
Randomization	Not relevant to SIOP-2001 study as the anaplastic Wilms tumor samples were of patients that were treated as standard of care. SIOP-2001 comprised a clinical trial for the stage II and stage III nephroblastoma of INTERMEDIATE risk and hence not anaplastic histology risk.
Blinding	Not relevant to this study as the clinical trial part involved only patients with stage II and stage III nephroblastoma with INTERMEDIATE risk and hence not anaplastic histology risk.

Reporting for specific materials, systems and methods

We require information from authors about some types of materials, experimental systems and methods used in many studies. Here, indicate whether each material, system or method listed is relevant to your study. If you are not sure if a list item applies to your research, read the appropriate section before selecting a response.

Materials & experimental systems

n/a	Involved in the study
[ ]	[x] Antibodies
[ ]	[x] Eukaryotic cell lines
[x]	[ ] Palaeontology and archaeology
[x]	[ ] Animals and other organisms
[ ]	[x] Clinical data
[x]	[ ] Dual use research of concern
[x]	[ ] Plants

Methods

n/a	Involved in the study
[x]	[ ] ChIP-seq
[x]	[ ] Flow cytometry
[x]	[ ] MRI-based neuroimaging

Antibodies

Antibodies used

CD3 DAKO Polyclonal ready to use
CD8 DAKO C8/144B 1/1000
EZH2 AbCAM ab191080 1/500

Validation

https://www.agilent.com/en/product/immunohistochemistry/antibodies-controls/primary-antibodies/cd3-%28dako-omnis%29-76197
https://www.agilent.com/en/product/immunohistochemistry/antibodies-controls/primary-antibodies/cd8-%28dako-omnis%29-76236
https://www.abcam.com/products/primary-antibodies/kmt6--ezh2-antibody-epr93072-n-terminal-ab191080.html

Eukaryotic cell lines

Policy information about cell lines and Sex and Gender in Research

Cell line source(s)

WT 17.94 cell line was chosen as was one of the rare anaplastic Wilms cell lines commercially available. The 17.94 cell line (cat no. ACC 741) was purchased from Leibniz-Institut DSMZ-Deutsche Sammlung von Mikroorganismen und Zellkulturen GmbH.

Authentication

The identity of the cell lines was confirmed by short tandem repeat (STR) analysis.

Mycoplasma contamination

All cell lines were regularly tested as negative for Mycoplasma infection using the Venor™ GeM Mycoplasma Detection Kit, and used at less than 10 passages.

Commonly misidentified lines
(See ICLAC register)

No lines of this category were used.

Clinical data

Policy information about clinical studies

All manuscripts should comply with the ICMJEguidelines for publication of clinical research and a completedCONSORT checklist must be included with all submissions.

Clinical trial registration

SIOP 2001

Study protocol

https://www.skion.nl/workspace/uploads/Protocol-SIOP-2001.pdf

Data collection

Clinical data collection was centralized.

Outcomes

The primary outcome for patients was overall survival.
